# Insights into the Folding and Unfolding Processes of Wild-Type and Mutated SH3 Domain by Molecular Dynamics and Replica Exchange Molecular Dynamics Simulations

**DOI:** 10.1371/journal.pone.0064886

**Published:** 2013-05-29

**Authors:** Wen-Ting Chu, Ji-Long Zhang, Qing-Chuan Zheng, Lin Chen, Hong-Xing Zhang

**Affiliations:** State Key Laboratory of Theoretical and Computational Chemistry, Institute of Theoretical Chemistry, Jilin University, Changchun, Jilin, People's Republic of China; Universitat Autònoma de Barcelona, Spain

## Abstract

Src-homology regions 3 (SH3) domain is essential for the down-regulation of tyrosine kinase activity. Mutation A39V/N53P/V55L of SH3 is found to be relative to the urgent misfolding diseases. To gain insight, the human and gallus SH3 domains (PDB ID: 1NYG and 2LP5), including 58 amino acids in each protein, were selected for MD simulations (Amber11, ff99SB force field) and cluster analysis to investigate the influence of mutations on the spatial structure of the SH3 domain. It is found that the large conformational change of mutations mainly exists in three areas in the vicinity of protein core: RT loop, N-src loop, distal β-hairpin to 3_10_ helix. The C-terminus of the mutated gallus SH3 is disordered after simulation, which represents the intermediate state of aggregation. The disappeared strong Hbond net in the mutated human and gallus systems will make these mutated proteins looser than the wild-type proteins. Additionally, by performing the REMD simulations on the gallus SH3 domain, the mutated domain is found to have an obvious effect on the unfolding process. These studies will be helpful for further aggregation mechanisms investigations on SH3 family.

## Introduction

Amyloids are insoluble β sheet-rich fibrous protein aggregates sharing specific structural traits [1]. Some misfolded structures of amyloids, mainly caused by site- directed mutagenesis, alter their proper configuration such that they erroneously interact with one another or other cell components forming insoluble fibrils. Generally, these misfolded structures associate with the pathology of more than 20 serious human diseases, including Alzheimer's and Parkinson's disease and type II diabetes [2]–[5]. Abnormal accumulation of amyloid fibrils in organs may lead to amyloidosis and play a role in various neurodegenerative disorders [6], [7].

Nowadays, growing awareness of the misfolded amlyoids seems to be leading to substantial new research and treatment alternatives [4]. Fyn, a tyrosine kinase of the Src family, plays a role in the regulation of intracellular calcium levels [8]. Beside the catalytic domain, the Fyn-like kinases contain two small, mutually unrelated, non-catalytic domains (SH2 and SH3 domains, Src-homology regions 2 and 3) [9], [10]. It has been suggested that the SH2 and SH3 domains function as molecular adaptors that mediate molecular recognition events among proteins involved in signal transduction pathways [9]. Lastly, fyn, but not other tyrosine kinases, appears to be up-regulated in a subset of neurons in Alzheimer's disease brain [11]. Then in 2004, Ollerenshaw and others [12] applied essential dynamics method to indicate that the unfolding intermediate was formed by dissociation of the folded protein's two terminal β strands from its core. Mutations and deletions of the SH3 domains in protein tyrosine kinases will result in the activation of the transforming potential of these proteins. This implies that SH3 domain plays an important role in the down-regulation of kinase activity either via the interactions with other regulatory proteins or via an intramolecular mechanism [13], [14]. According to the investigation of wild type and mutated Spc-SH3 domain, the homologous protein of Fyn-SH3 domain by Krobath et al. [15], the β1 strand was totally unstructured and residues 44 and 53 were also important triggers of the aggregation process. Therefore, mutations on some conserved residues of SH3 domain will influence the regular folding process, leading to protein misfolding diseases.

**Figure 1:**
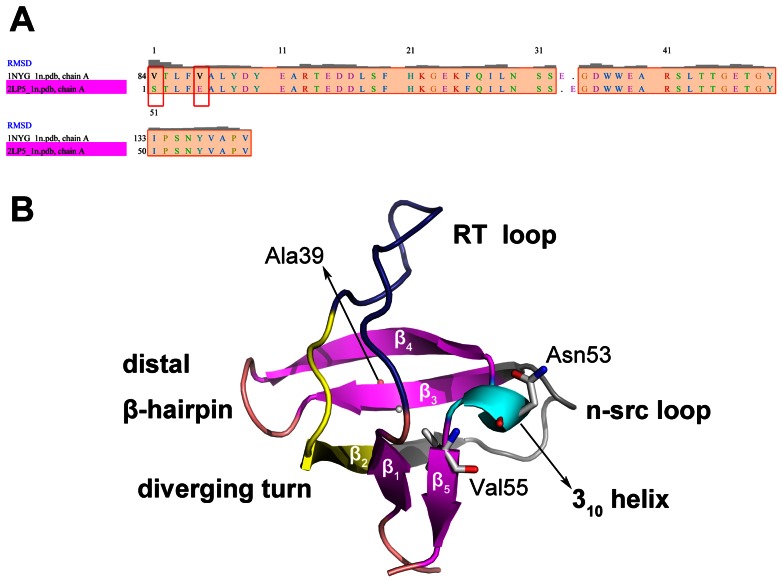
SH3 domain sequence and structure. Sequence alignment of the human (1NYG) and gallus (2LP5) SH3 domain (A) is generated by Chimera 1.5.3 [42]. Only residue 1 and residue 5 between the two sequences are different. In crystal structure of the gallus SH3 domain (PDB ID: 2LP5) (B), the SH3 domain is divided into several parts, such as RT loop, diverging turn, N-src loop, distal β-hairpin, 3_10_ helix, and five strands. Mutation sites, Ala39, Asn53, and Val55 are shown in white sticks and labeled in the figure. All the oxygen atoms and nitrogen atoms of these residues are colored red and blue, respectively.

The crystal structure of SH3 domain (Val84 to Asp142) was firstly obtained by Noble et al. in 1993 [8]. As shown in Figure 1, the SH3 domain is generally divided into five structural regions: N- and C-terminal strands, RT loop (residues 8–19, Val84 as residue 1, same as below), diverging type II β-turn (residues 20–27), n-src loop (residues 28–37), and distal β-hairpin (residues 42–45) [16]. The SH3 domain has a characteristic β-barrel fold which consists of five β-strands arranged as two tightly packed anti-parallel β sheets. The anti-parallel β sheets are linked with a short 3_10_ helix. Chiti and Jahn et al. reported that the formation of fibril was also observed for globular proteins under native conditions, and aggregation proceeded via native-like intermediates that are formed after the major folding barrier in many of these systems [5], [17]. In the previous NMR studies by Neudecker et al. [18], [19], they demonstrated that the A39V/N53P/V55L mutant SH3 domain folds from the unfolded state (U) via an on-pathway, low-populated intermediate (I) to native β-sandwich folds (N). In addition, the NMR results of Korzhnev et al. [20] revealed that the I states of the G48M and G48V mutants differ significantly in their structural heterogeneity, suggesting the crucial role of position 48 in the folding of Fyn SH3. This I state and fibril formation, as well as the identification of specific structural elements that can be targeted through rational design of therapeutics against protein misfolding diseases, such as the Alzheimer's disease. As a result, the research on the A39V/N53P/V55L mutant is helpful to detect the I state and find out the relationship between mutation and protein misfolding diseases. Most notably, newly obtained NMR structure of gallus SH3 domain by Neudecker et al. [21] elucidated that the C-terminus from Pro57 to Asp59 is disordered in I, which implies that strand β5 was not formed. However, there still lacks the detailed dynamic and mutated studies at atomic level of SH3 domain folding and unfolding process.

So far, numerous of crystal and NMR structures of Fyn SH3 domains have been deposited into Protein Data Bank [8], [21]–[25]. In the current study, we focus on the folding and aggregation processes of the human and gallus Fyn SH3 domains by using mutation studies, molecular dynamics (MD) simulations, and replica exchange molecular dynamics (REMD) method. MD simulations can provide useful means to obtain atomic level views of protein unfolding not accessible to direct experimental measurement [26]. The REMD method is used to enhance sampling relative to a standard molecular dynamics simulations by allowing systems of similar potential energies to sample conformations at different temperatures [27]. Here we report the MD and REMD simulation results of human and gallus Fyn SH3 domains combined with their mutants, which may be helpful for further protein folding and unfolding mechanism studies.

## Materials and Methods

### 1. Initial Model of Systems

The atomic coordinates and the structure factors for the NMR structures of wild-type *Homo sapiens* and *Gallus gallus* Fyn SH3 domain have been deposited in the Protein Data Bank (PDB ID: 1NYG [22] and 2LP5 [21]), including 58 amino acids. The native states of 1NYG (Val1 to Val58) and 2LP5 (Ser1 to Val58) were selected as system 1 and system 2 (known as **hn** and **gn**) for MD simulations. In addition, two mutant models of the conserved residues, A39V/N53P/V55L, were selected as system 3 and system 4 for MD simulations (known as **hm** and **gm**). These two systems were prepared by Swiss PDB Viewer software [28]. All missing hydrogen atoms of the proteins were added using the LEaP module in the AMBER 11 package [29]. The ff99SB force field [30] was applied to produce the parameters for the protein. Finally, an appropriate number of sodium counterions were placed in the complex systems, which were solvated in a truncated octahedral periodic box of TIP3P water [31] molecules with a minimum distance of 10 Å between the outermost protein atoms and the walls of the simulation box.

### 2. Molecular Dynamics Simulations

Four systems were prepared for the MD simulations: native state *Homo sapiens* Fyn SH3 domain (**hn**); native state *Gallus gallus* Fyn SH3 domain (**gn**); mutated state *Homo sapiens* Fyn SH3 domain (**hm**); mutated state *Gallus gallus* Fyn SH3 domain (**gm**). Two steps energy minimizations were carried out on all the systems, using SANDER module of AMBER 11 [32]. First, all the water molecules and counterions were minimized with 2000 steps of steepest descent followed by 3000 steps of conjugate gradient. Then the systems were minimized with the same process to remove the bad contacts. Then we tested the quality of the two initial structures of the **hn** and **gn** runs. Ramachandran plot of them was shown in the Figure S1, using the on-line tool Molporbity [33]–[35]. 92.7% and 100.0% of all the residues in **hn** system were in the favored and allowed regions, 96.4% and 100.0% of all the residues in **gn** system were in the favored and allowed regions, respectively. The results have proved that the initial models of the MD simulations are all reasonable. The four systems were gradually heated up to 300 K in the NVT ensemble, with velocity of 1 K/ps. Then, they reached equilibration after another 200 ps simulation. Meantime, weak restrains were performed on the Cα atoms of protein during the first two processes to ensure the accomplishment of the stabilization. Finally, 200 ns MD simulation was carried out on each system with the NPT ensemble. SHAKE algorithm [36] was applied to constrain all bonds involving hydrogen atoms, and particle mesh Ewald (PME) method [37] was used to calculate the electrostatic interactions with a cutoff value of 10 Å. The time step was 2 fs.

### 3. Cross-correlation Analysis

Cross-correlation analysis was used to investigate the extent of correlation motions during the four complex trajectories. Generally, the cross-correlation matrix, *C_ij_*, was calculated to reflect the Cα atoms fluctuations relative to their average coordinates. All the 200 ns simulations of the four systems were selected to the calculations by the following equation:
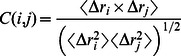
(1)In eq 1, the angle bracket represents an average over the sampled period and Δ*r_i_* indicates the deviation of the Cα atom of *i*th residue from its mean position [38]. The value of *C_ij_* fluctuates from −1 to 1. Positive *C_ij_* values represent a correlated motion between the *i*th residue and the *j*th residue, while the negative values describe an anti-correlated motion.

### 4. Cluster Analysis

Clustering is a general data-mining technique that can be applied to any collection of data elements (points) where a function measuring distance between pairs of points is available [39]. In this contribution, module ptraj of AMBER 11 [29] was used for the cluster analysis of the four trajectories. We performed the cluster analysis to produce 5 clusters using the pairwise root mean square deviation (RMSD) between frames as a metric comparing the atoms named CA, implemented the averagelinkage algorithms [40]. The structures were optimally superposed prior to the RMSD, and the representative structures were used as the reference structure. 20000 frames of all the 100000 frames were used for the analysis of each system. Other settings of cluster calculations were used as default.

### 5. Replica Exchange Molecular Dynamics method

REMD method can effectively sample high-dimensional rough energy landscapes, especially valuable for the research of protein folding and unfolding processes. Owing to the computational resource limitation, we only performed the REMD simulations of the **gn** and **gm** systems, using sander module of AMBER 11. After minimization and heat steps, the last frame of the heated structure was subjected to the 200 ps equilibration step, with the target temperature of 280, 295, 310, 325, 340, 355, 370, 385, 400, 415, 430, 445 K, resulting in 12 replicas. The REMD was simulated on each replica for 100 ns, resulting in a total simulation time of 2400 ns. The generalized Born (GB) model [41] was used for the two REMD runs in order to mimic the solvation effect. Other settings of REMD simulation were applied as the normal MD simulation.

## Results and Discussion

### 1. MD simulations on human and gallus SH3 domains

200 ns MD simulations were carried out on the four systems (**hn**, **hm**, **gn**, and **gm**) to study the conformational change on each protein. The calculated total energy and potential energy for all the systems were shown in Figure S2A and S2B. The energy results indicate that all the four systems have reached equilibration, which proves that the MD simulation method for this kind of protein is reliable. In addition, it is clear to find out in Figure S2 that the energy increment from mutated to native gallus SH3 domain increases a bit more than that from mutated to native homo SH3 domain, which means that the mutations may make a bigger impact on the gallus SH3 domain.

**Figure 2:**
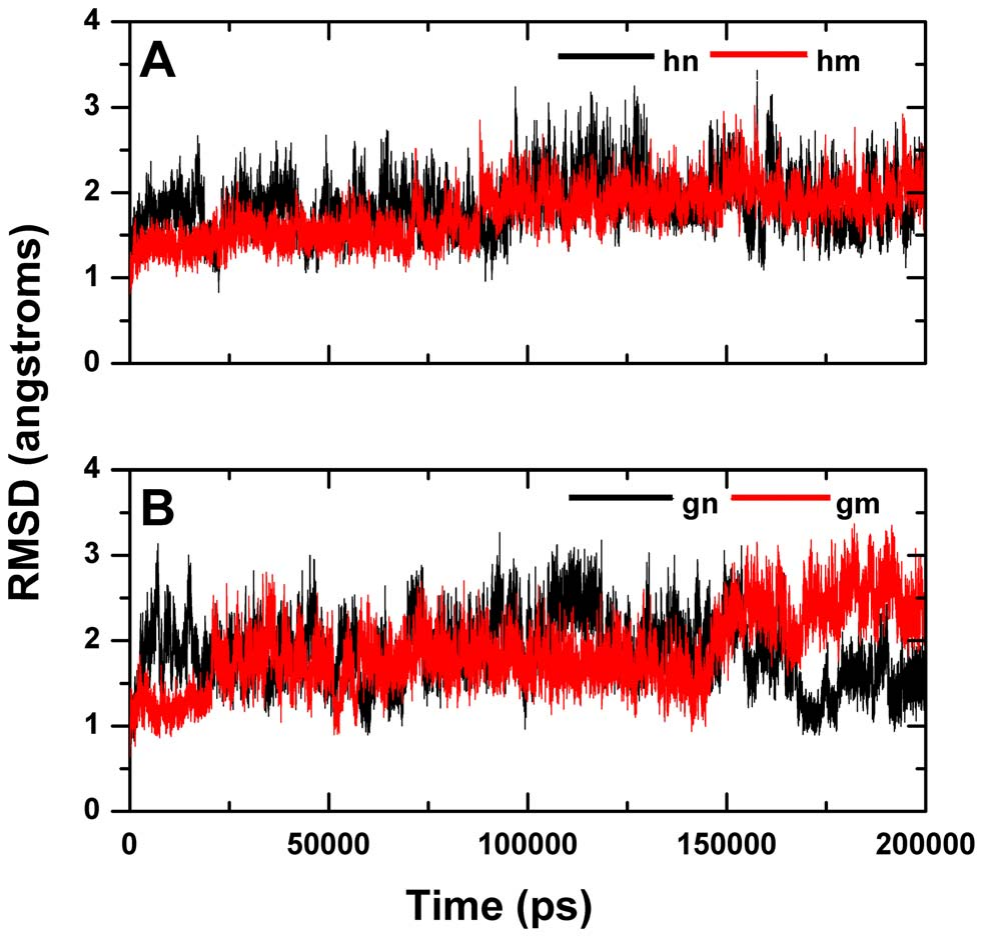
The RMSD curves of the four 200 ns trajectories. RMSD values of the protein backbone atoms of human (A) and gallus (B) SH3 domains are calculated with respect to the crystal structure as a function of simulation time for wild-type proteins (black) and A39V/N53P/V55L mutations (red), labeled as **hn**, **hm**, **gn**, and **gm**.

In the case of human SH3 domain, it is easy to find that the backbone root mean square deviation (RMSD) values of both the **hn** and **hm** systems are stabilized at around 2.0 Å (Figure 2A). The RMSD values of **hm** are higher than that of **hn** for about 0.2 Å at the end of trajectories. However, conditions get different in the case of gallus SH3 domain. The RMSD values of **gn** system is equilibrated at about 1.5 Å, but those of **gm** system reach nearly 2.5 Å at the end of the simulations (Figure 2B). In summary, all the mentioned above illustrates that mutation on A39V/N53P/V55L of gallus SH3 domain will result in large conformational change of protein.

**Figure 3:**
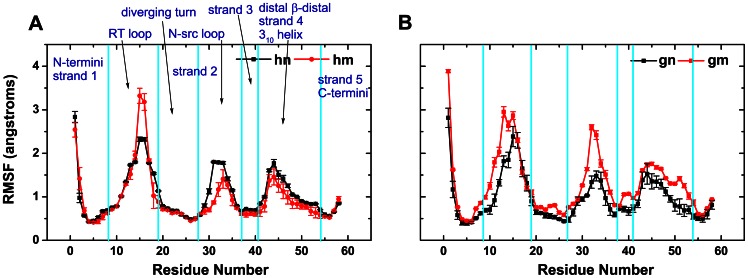
The RMSF curves of the protein backbone atoms relative to the crystal structure for the four systems, colored as Figure 2. The SH3 domain includes β1 (residues 3–7), RT loop (residues 8–19), diverging turn (residues 20–27), N-src loop (residues 28–37), β2 (includes part of diverging turn and N-src loop, residues 25–30), β3 (residues 38–41), distal β-hairpin (residues 42–45), β4 (residues 46–50), 3_10_ helix (residues 51–54), and β5 (residues 55–57). Each part of SH3 domain and error bars of RMSF are labeled in this figure.

As Riddle et al. [16] pointed out, the SH3 domain could be divided into five structural regions according to the importance in the folding transition state: N- and C-terminal strands and 3_10_ helix, RT loop, diverging turn, N-src loop, and distal β-hairpin (shown in Figure 1B). In detail, the content of all the parts are described as follows: β1 (residues 3–7), RT loop (residues 8–19), diverging turn (residues 20–27), N-src loop (residues 28–37), β2 (includes part of diverging turn and N-src loop, residues 25–30), β3 (residues 38–41), distal β-hairpin (residues 42–45), β4 (residues 46–50), 3_10_ helix (residues 51–54), and β5 (residues 55–57). After simulations, root mean square fluctuation (RMSF) was measured to investigate the effect of mutation on different areas of the protein during the MD simulations, see Figure 3A and 3B. In the case of human SH3 domain, three areas (RT loop, N-src loop, distal β-hairpin to 3_10_ helix) have large RMSF values. It should be noticed that the RT loop (residue 8 to 19) of the **hm** system has large conformational fluctuations than that of the **hn** system during the simulation. The mutation of human system mainly influences the structure of RT loop region, but the mutation of gallus system will result in large RMSF values on almost all the **gm** protein. Interestingly, the three areas of human SH3 domain which have large RMSF values are in the vicinity of the protein center. However, diverging turn and strand 3 of the gallus SH3 domain, which are near the protein center, also have larger RMSF values in the mutated SH3 compared to the wild-type SH3. Therefore, we could conjecture that the large conformational fluctuations on both central and peripheral protein which attribute from the mutations would affect the protein folding process of SH3 domain.

### 2. Cross-correlation Analysis

**Figure 4:**
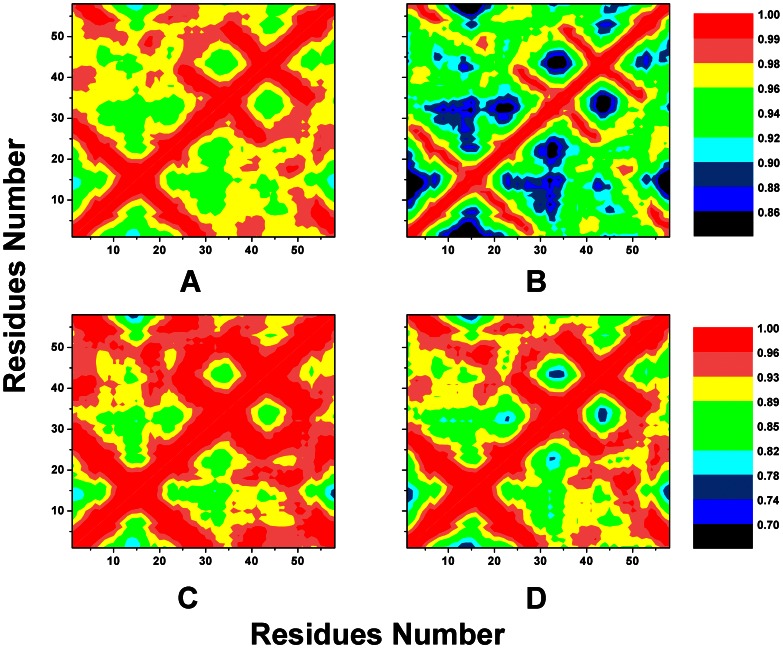
Cross-correlation matrices of the coordinate fluctuations. The Cα atoms of systems **hn** (A), **hm** (B), **gn** (C), and **gm** (D) are calculated around their mean positions during the 200 ns simulations. The extents of residues motions are labeled in different colors.

Cross-correlation analysis was used to study the residue changes in the internal motions induced by the mutated SH3 domain. It is clear in Figure 4 that almost all the interactions of wild-type simulation are filled with higher positive correlation movements. These movements are shown in red, wine and yellow colors. By contrast, some internal interactions change lower positive correlation motions under the influence of mutations. The motions are colored with blue, dark blue and black, see Figure 4.

Overall, the mutation of gallus SH3 domain has a larger effect on the protein structure as compared with that of the human SH3 domain (data not shown in Figure 4). In the case of human SH3 system, it is easy to find out that lower positive correlation motions mainly appear at the RT loop, N-src loop, distal β-hairpin to 3_10_ helix regions, just as the three regions mentioned above. In the case of gallus SH3 system, same areas have lower positive correlation motions. The corss-correlation results suggest that the internal motions of SH3 domains are significantly affected by both the two mutations. The protein tends to be looser in the mutations, as a result of moving away of loop regions and 3_10_ helix from the protein center.

### 3. Cluster Analysis

**Figure 5:**
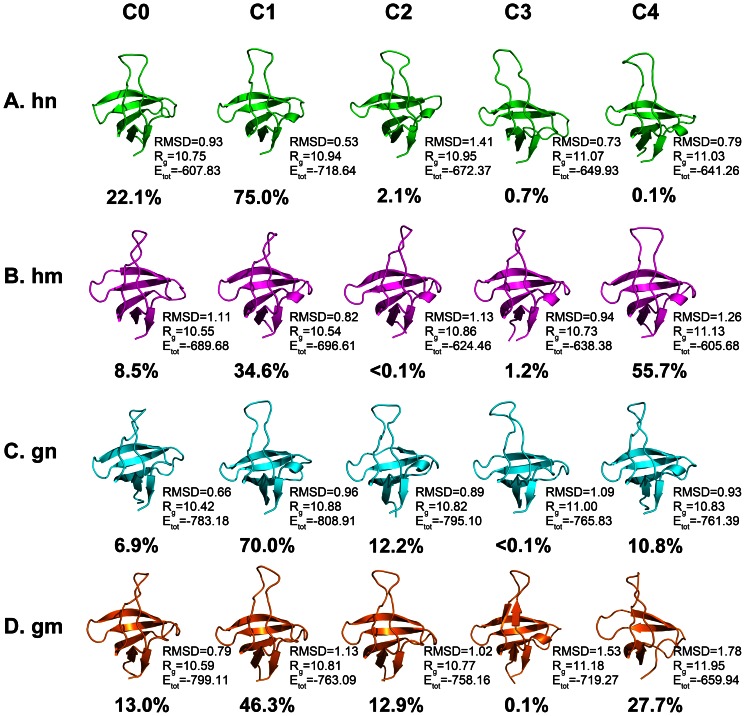
Cluster analysis for the simulations of the four systems. All the representative structures of **hn** (A), **hm** (B), **gn** (C), and **gm** (D) are shown in cartoon and colored by green, magenta, cyan, and orange, respectively. The occurrence of each cluster is labeled in this figure. RMSD respected to the crystal structure, radius of gyration (R_g_), and total energy (E_tot_) of the protein in each cluster are added in this figure, and the units of them are Å, Å, and Kcal/mol, respectively.

Cluster analyses for the four systems were carried out by the ptraj module of AMBER11. Each trajectory was divided into five clusters (C0, C1, C2, C3, and C4) according to the RMSD values, as shown in Figure 5. It is clear that mutations of both human and gallus SH3 domain have affected the protein folding process.

Firstly, we focus on the human SH3 system. In the **hn** system, cluster C0 and C1 (22.1% and 75.0%) almost cover all the structures in the trajectory (Figure 5A). The representative structure of cluster C0 shows that the 3_10_ helix starts to disorder, but other parts of C0 are similar to that of C1. When protein is mutated, the trajectory of mutation A39V/N53P/V55L obviously changes from that of the native state. As shown in Figure 5B, cluster C4 (55.7%) and C1 (34.6%) take an important part in the **hm** trajectory. But both clusters C1 and C4 have conformational change on the RT loop, which causes the changes of the protein shape. Additionally, the radius of gyration (R_g_) of the crystal structure (1NYG), cluster C1 of **hn**, and cluster C4 of **hm** is 10.73 Å, 10.94 Å, and 11.13 Å, respectively. Judging from the results of the R_g_, we can easily find the volume changes of the mutated protein. The difference on the flexible RT loop of the two high-populated clusters of **hm** are evaluated by distance between the α carbon atoms of Thr14, Glu15, and Asp16, as shown in Figure 3A. But all the five β strands of them are similar. This part of RT loop is rich of polar amino acids, and the largest length (Glu15/C1 to Glu15/C4) is 7.7 Å, which changes the shape of RT loop and makes it move forward at the latter part of the MD simulation. Overall, the volume of the mutated human SH3 domain is a bit larger than the native one.

The cluster analysis results of the gallus SH3 system have something different from that of the human SH3 system. In the **gn** system, cluster C1 (70.0%) occupies most frames of the trajectory (Figure 5C). In the **gm** system, cluster C1 (46.3%) exists in the first half of the trajectory. However, in the latter part of **gm** trajectory, cluster C4 (27.7%) is found to have large conformational change on both secondary and tertiary structures. Because all the clusters are arranged in chronological order of the MD runs, the cluster C4 of the **gm** system represents the structures of mutated gallus SH3 domain during the last 50 ns of the MD trajectory. It is clear to observe that the β5 strand and 3_10_ helix are completely disordered in C4. Then we have calculated the secondary structure content of the 2LP5 and the representative structure of cluster 4. In 2LP5, the secondary structure includes five strands, which are residue 3–7, 25–30, 37–41, 47–51, and 55–57. In our simulation, the cluster 4 includes four strands (β1 4–7, β2 25–28, β3 38–40, and β4 47–50). The length of the β2, β3, and β4 has been shorten, which means that the SH3 domain tends to be partly disordered in the latter part of **gm** trajectory. Normally, β1 and β5 tightly packed into an anti-parallel β sheet. But this sheet is disappeared in C4 with the disordered β5. Additionally, the R_g_ of the crystal structure (2LP5), cluster C1 of **gn**, and cluster C4 of **gm** is 10.63 Å, 10.88 Å, and 11.95 Å, the energy of them is −784.89 Kcal/mol, −808.91 Kcal/mol, and −659.94 Kcal/mol. And the RMSD of C4 respect to the 2LP5 is 1.78 Å. These results indicate that the mutated gallus SH3 domain has experienced a large conformational change, leading to larger volume and a disordered C-terminus. The structures of C1 and C4 of **gm** system are aligned in Figure S3B. Apart from the changes of β strands, almost all the loops have large conformational changes during the MD simulation. By calculating the distance between the α carbon atoms of Thr14, Ser32, Thr44, and Ser52, we can find out that the RT loop is moving forward, while the other three parts of loop are moving backward. These movements will cause the shape of the mutated gallus SH3 domain to be larger.

Aforementioned experimental results of mutated gallus SH3 domain (A39V/N53P/V55L) by Neudecker et al. [21] demonstrated that the low populated intermediate state (I) had disordered C-terminus from Pro57 to Asp59, which is consistent with the structure of C4 in **gm** system. That means, during the last 50 ns of the **gm** MD simulation, some SH3 domains in native state tend to switch to the intermediate state (I). But that phenomenon is not found in the human SH3 domain. Figure S4A shows the secondary structure content of the last 50 ns of the four systems, which also support that point. Though it seems that the C-terminus is not disordered during the **hm** simulation, corss-correlation results prove that the shape of the **hm** protein tends to be changed after simulation, just as that of the **gm** protein. Figure S3C shows the comparison of the most-populated clusters of four systems, C1 of **hn**, C4 of **hm**, C1 of **gn**, and C4 of **gm**, with the three mutation sites labeled, residue 39, 53 and 55. The C4 of **gm** has the largest conformational change. It is clear to find out the residue 39 of **gm** have an effect on the length of the β2, β3, and β4, also, the residue 53 and 55 cause the disorder of 3_10_ helix and β5. Furthermore, we calculated the mean solvent accessible surface area (SASA) per residue of each system compared with the crystal structures (Figure S4B). The hydrophobic residues focus on the C-terminus of the SH3 domain, and the SASA of Tyr49, Pro51, Ala56, Pro57, and Val58 has markedly increased in the **gm** system. In addition, some hydrophobic core residues, Phe20, Phe26, and Trp36 of the **gm** system also have larger SASA values. To conclude, the effect of mutation A39V/N53P/V55L on the folding process of gallus SH3 system would be much bigger than that of human SH3 domain.

### 4. Hbond net of SH3 systems

**Figure 6:**
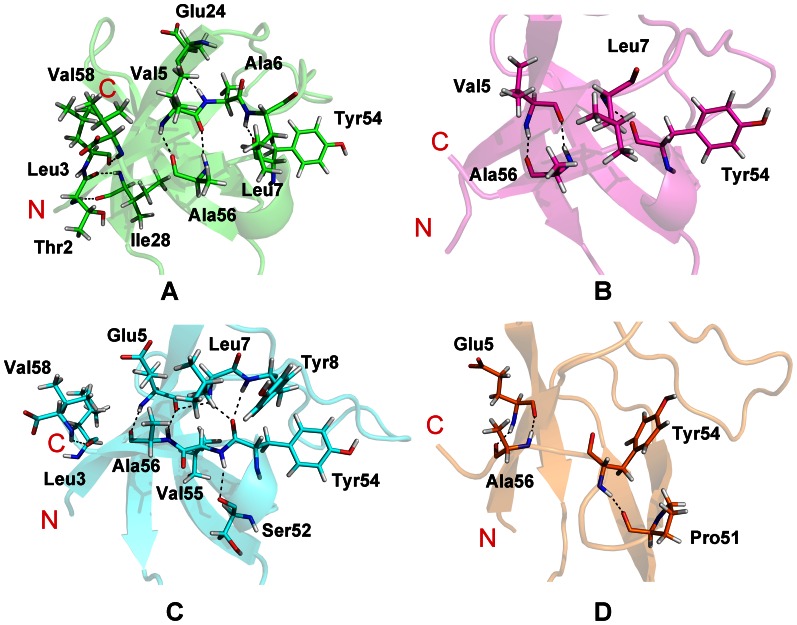
The representative structures of cluster C1, C4, C1, and C4 for the systems. Each system, **hn** (A), **hm** (B), **gn** (C), and **gm** (D), is colored as Figure 5. The Hbonds are labeled in black dash line. All the oxygen atoms are colored in red and nitrogen atoms are colored in blue.

Aiming to find out the reason of folding process changes by mutations, the Hbonds in each system are represented in Figure 6. The distance and the angel of Hydrogen bonds (Hbonds) calculations were set to 3.5 Å and 120°, respectively. To clearly show the interactions within the proteins, we focus on the cluster C1, C4, C1, and C4 of the systems **hn**, **hm**, **gn**, and **gm**, respectively.

It should be noticed that there exists a strong Hbond net in the native state SH3 domain. But this net is nearly destructed under the influence of the mutations A39V/N53P/V55L. In the human SH3 system, the Hbonds between Val5 and Ala56, between Leu7 and Tyr54 are conserved in both native and mutated protein. But some Hbonds on the N- and C-terminal strands and 3_10_ helix, such as the Hbonds around Leu3, Ala 6, and Val58 are destroyed in the **hm** protein. In the case of gallus SH3 system, the Hbond between Leu7 and Tyr54 is disappeared in the **gm** protein. With the movement of 3_10_ heliex, the hydrogen atom of Tyr54 forms an Hbond with the oxygen atom of Pro51. Because of the disordered C-terminal strand, the Hbond between Val58 and Leu3 is disrupted. Though the fifth residue is different between human and gallus systems, the backbone hydrogen atom and oxygen atom of this residue can form two Hbonds with the oxygen atom and the hydrogen atom of Ala56, respectively. In conclusion, mutations of SH3 proteins will disrupt some important Hbonds around the N- and C-terminal strands and 3_10_ helix, which is consistent with the conclusion that mutations will cause the change of the protein shape.

### 5. REMD simulations on gallus SH3 domain

REMD method can help proteins to get over the energy barrier and reach the equilibrated state during the folding and unfolding processes, by the exchange between lower temperature and higher temperature. Because the mutated gallus SH3 domain has more obvious effect on the folding process than the native one, **gn** and **gm** systems are selected for two parallel REMD runs (12 replicas each, 280 K to 445 K).

**Figure 7:**
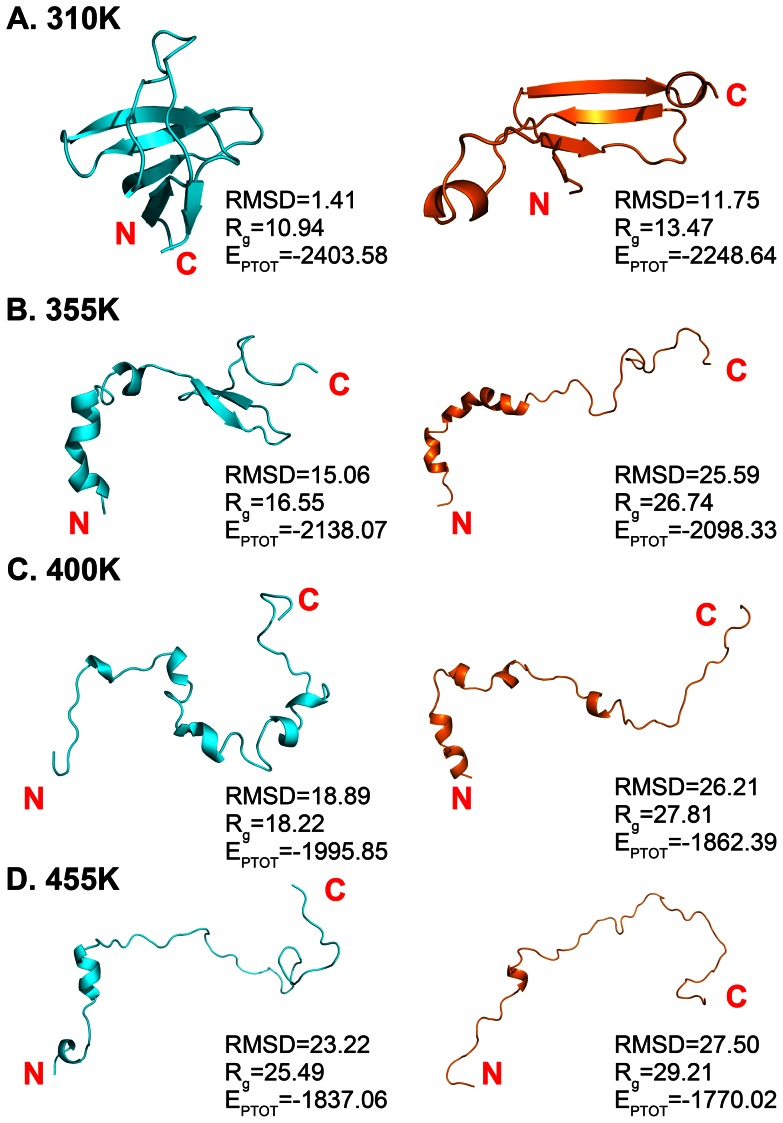
The representative structures of temperature 310 (A), 355 (B), 400 (C), and 455 K (D) for gn and gm systems. The systems **gn** and **gm** are colored with cyan and orange. RMSD respected to the crystal structure, radius of gyration (R_g_), and potential energy (E_PTOT_) of the protein in each cluster are added in this figure, and the units of them are Å, Å, and Kcal/mol, respectively.

After the REMD runs, the 12 replicas were transformed into 12 trajectories with different temperatures. Then we focus on the temperature 310 K and 400 K, the physiological temperature and the temperature that the β sheets have been completely disappeared. The RMSD values of the two temperatures respected to the crystal structure are shown in Figure S5. The RMSD result at 310 K of **gn** is stabilized below 2 Å, but that of **gm** is about 10 Å at the last half part of REMD run. In addition, the secondary structure content of **gm** at 310 K has decreased. As shown in Figure 7, the β5 at the C-teimini is already disordered and the RT loop moves a lot, but the core β strands, β2, β3, and β4 do not change dramatically, only the length of β2 decreased in **gm**. When the temperature increased to 400 K, the **gn** structure has been totally disordered except for some core areas, with higher than 15 Å RMSD values. However, at the same temperature, the **gm** structure has more disordered areas and shapes as a line, its RMSD is fluctuated during the trajectory and has reached nearly 27 Å at the end. (Figure 7 and S5) Additionally, potential energy (E_PTOT_) and R_g_ are calculated for gn and gm on each temperature, shown in Figure 7 (the kinetic energy changes with the temperature, as a result potential energy is used for comparison). The E_PTOT_ and R_g_ values increase as the temperature goes higher, and the results have also proved that the mutations have an obvious effect on the folding and unfolding processes of the gallus SH3 domain.

## Conclusion

Misfolded amyloids, associated with a wide range of human pathologies, are mainly caused by site-directed mutagenesis. SH3 domain has an important role in the down-regulation of tyrosine kinase activity. In this contribution, we mainly concern about how the mutation A39V/N53P/V55L of SH3 domain affects the normal folding process of this protein.

200 ns MD and 100 ns REMD simulations were carried out to investigate the influence of mutations. Our study demonstrates that the large conformational change of **hm** and **gm** mainly exists in three areas: RT loop, N-src loop, distal β-hairpin to 3_10_ helix, which are in the vicinity of protein center. The C-terminus is disordered during the **gm** simulation, which represents the intermediate state of aggregation. Additionally, the R_g_ results prove that mutations make the volume of SH3 protein to be larger, leading to the disruption of some important Hbonds. It should be noted that the strong Hbond net around the N- and C- termini of **hn** and **gn** systems is disappeared in the mutated systems. High temperature will cause the disappearance of protein secondary structure, but at the same temperature, the mutations will remarkly affect the regular folding process of gallus SH3 domain. Consequently, finding the effect of mutation A39V/N53P/V55L on SH3 folding process will pave the way for further aggregation mechanisms investigations on SH3 family.

## Supporting Information

Figure S1 Ramachandran plot of the two initial MD structures of the **hn** (A) and **gn** (B) systems.
(TIF)

Figure S2 The total energy (A) and potential energy (B) curves of the four 200 ns trajectories. These curves are calculated as a function of simulation time for wild-type proteins (black) and A39V/N53P/V55L mutations (red).
(TIF)

Figure S3 The comparison of the clusters C1 (green) and C4 (magenta) of **hm** system (A), C1 (cyan) and C4 (orange) of **gm** system (B), and the most-populated clusters of the **hn**, **hm**, **gn**, and **gm** systems (C). Some residues with large changes and the distance of them between these systems are labeled in this figure. The mutation sites are colored white.
(TIF)

Figure S4 **The secondary structure content and the mean solvent accessible surface area (SASA) figure.** The secondary structure content of the last 50 ns (A) is calculated for each system. And the SASA value per residue of each system (B) is compared with the crystal structures.
(TIF)

Figure S5 **The RMSD curves of the two REMD runs.** The protein backbone atoms RMSD values of **gn** (black) and **gm** (red) SH3 domains with respect to the crystal structure as a function of simulation time are compared at the temperature 310 K (A) and 400 K (B).
(TIF)
