# Supplementary figures and images for: Insights into the Folding and Unfolding Processes of Wild-Type and Mutated SH3 Domain by Molecular Dynamics and Replica Exchange Molecular Dynamics Simulations
Source: PLoS One. 2013 May 29;8(5):e64886. doi: 10.1371/journal.pone.0064886 (PMC3667132; doi:10.1371/journal.pone.0064886)

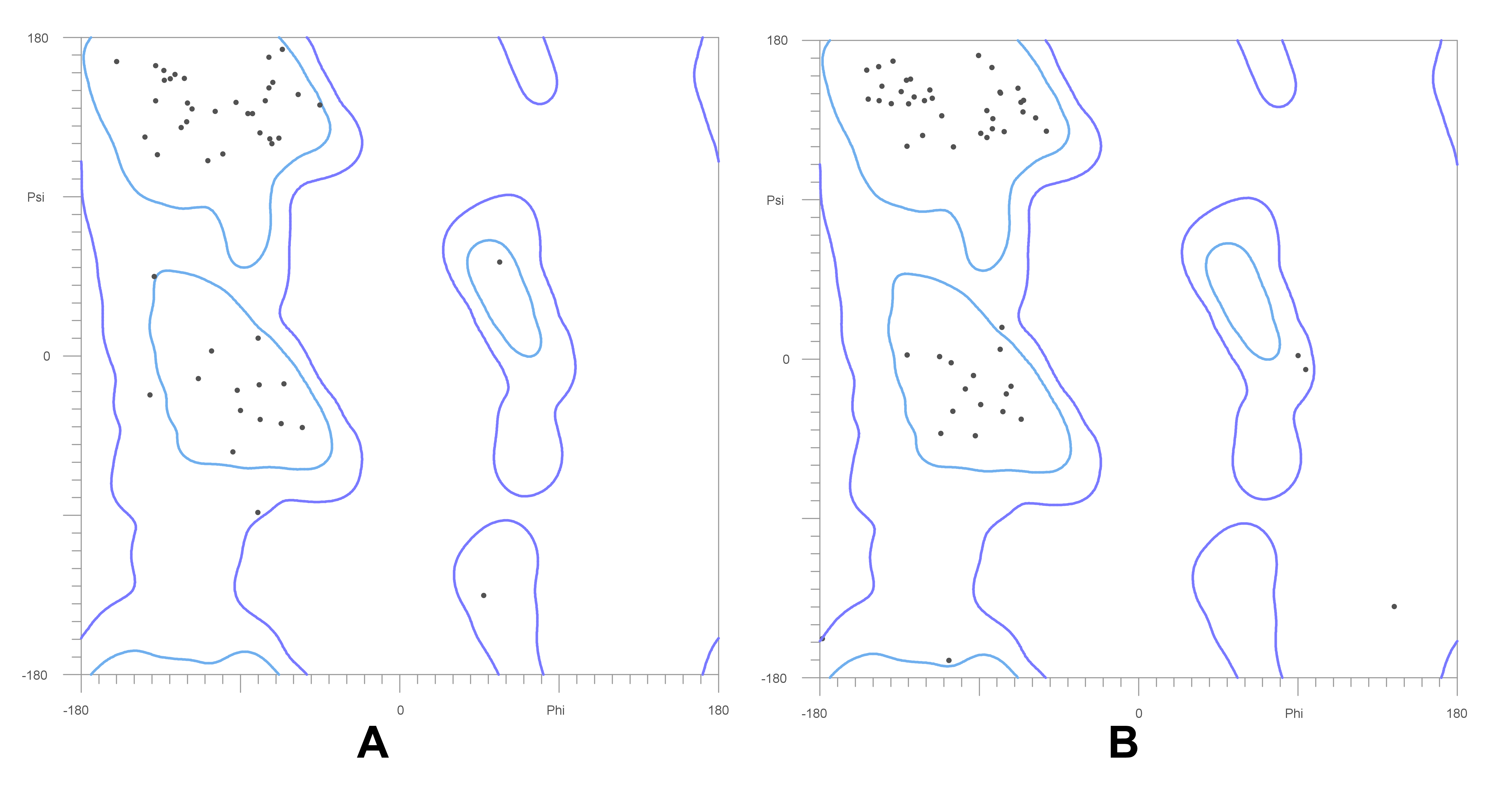

Supplement: Figure S1 — Ramachandran plot of the two initial MD structures of the hn (A) and gn (B) systems. (TIF) [file pone.0064886.s001.tif]

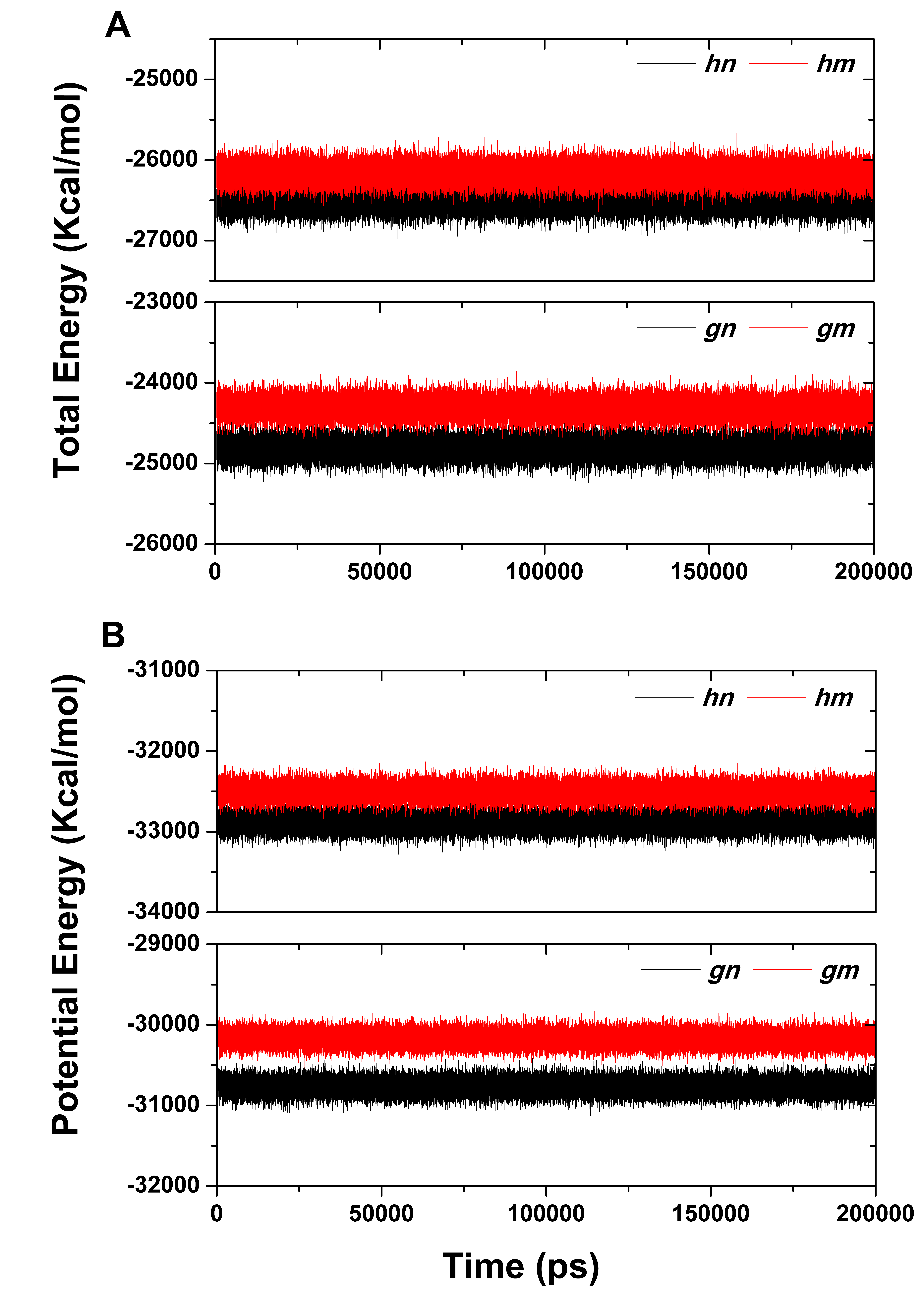

Supplement: Figure S2 — The total energy (A) and potential energy (B) curves of the four 200 ns trajectories. These curves are calculated as a function of simulation time for wild-type proteins (black) and A39V/N53P/V55L mutations (red). (TIF) [file pone.0064886.s002.tif]

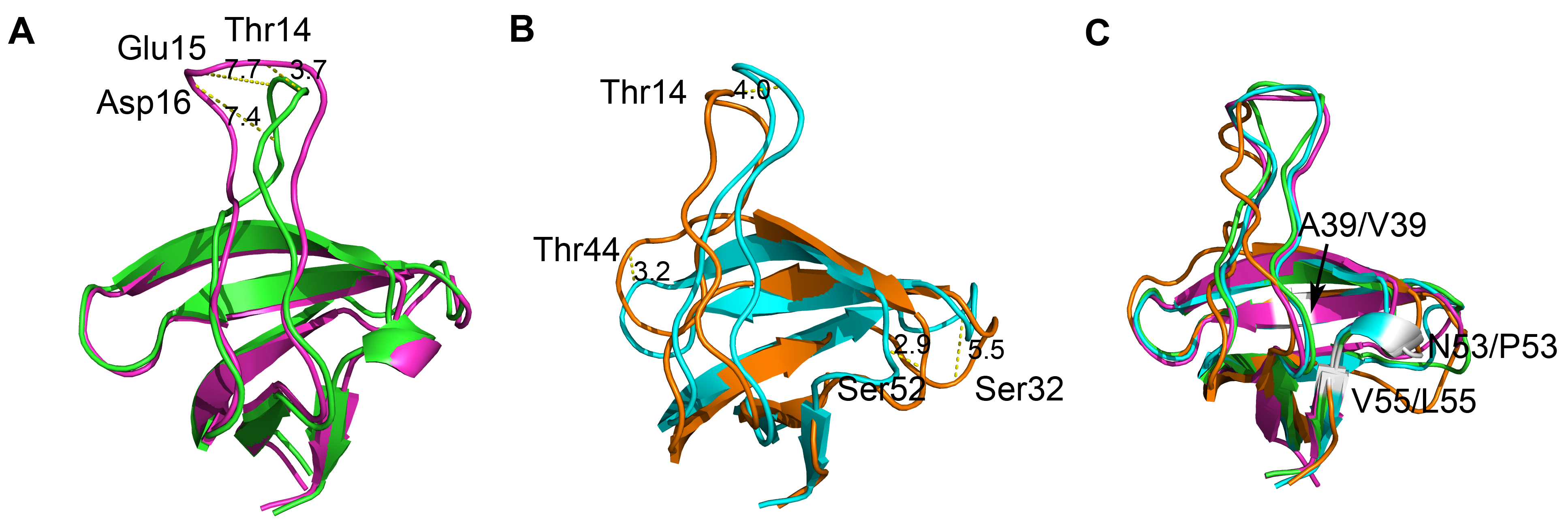

Supplement: Figure S3 — The comparison of the clusters C1 (green) and C4 (magenta) of hm system (A), C1 (cyan) and C4 (orange) of gm system (B), and the most-populated clusters of the hn, hm, gn, and gm systems (C). Some residues with large changes and the distance of them between these systems are labeled in this figure. The mutation sites are colored white. (TIF) [file pone.0064886.s003.tif]

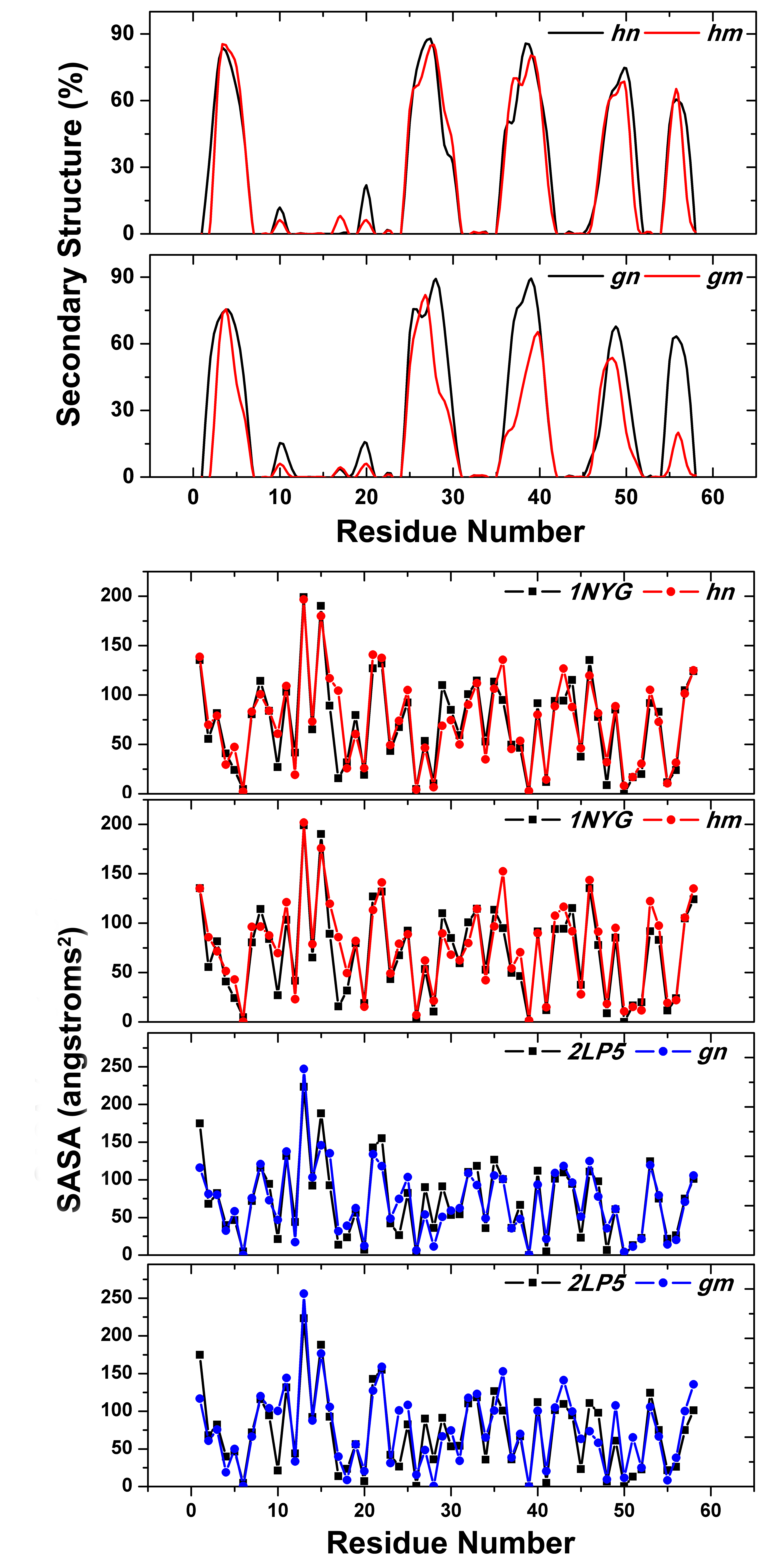

Supplement: Figure S4 — The secondary structure content and the mean solvent accessible surface area (SASA) figure. The secondary structure content of the last 50 ns (A) is calculated for each system. And the SASA value per residue of each system (B) is compared with the crystal structures. (TIF) [file pone.0064886.s004.tif]

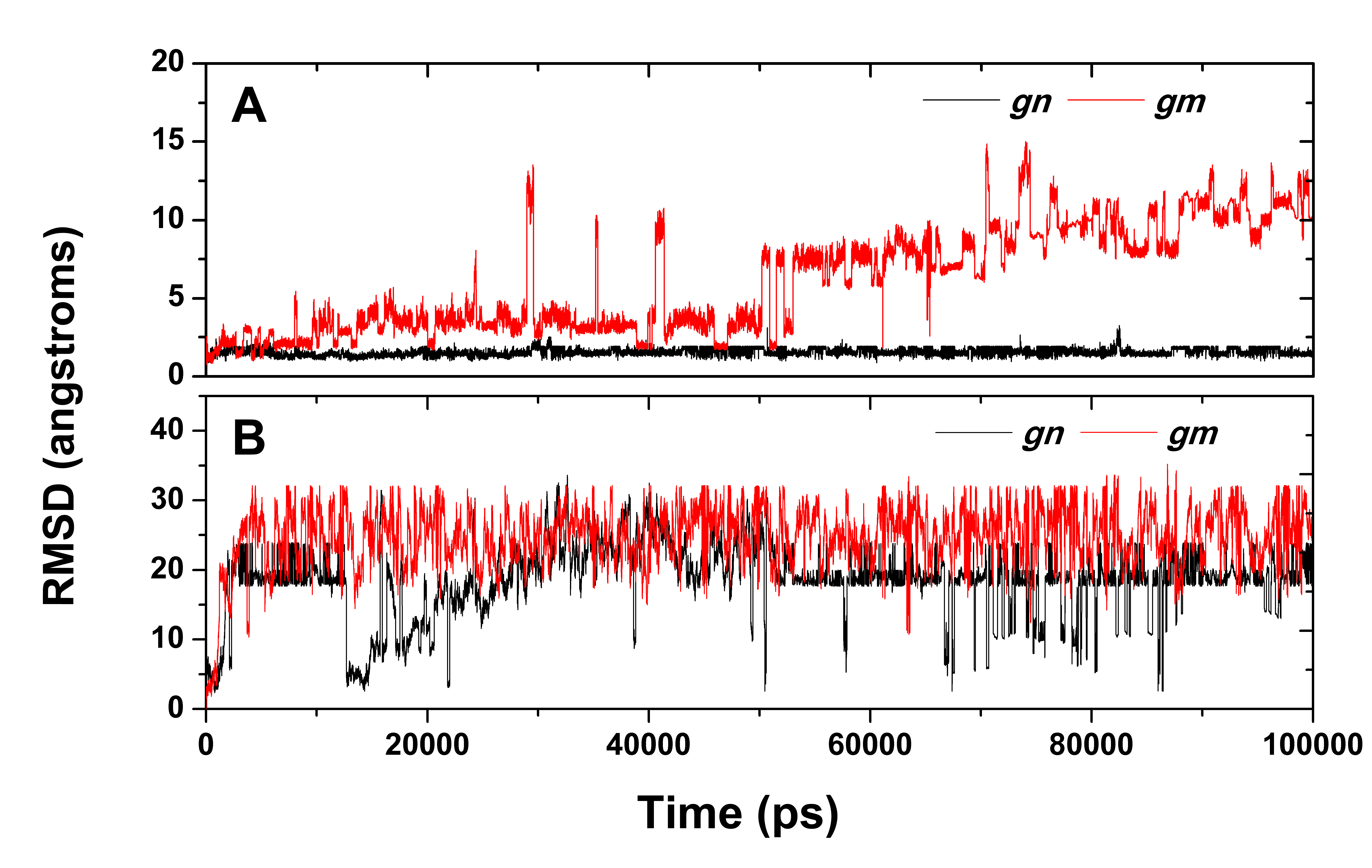

Supplement: Figure S5 — The RMSD curves of the two REMD runs. The protein backbone atoms RMSD values of gn (black) and gm (red) SH3 domains with respect to the crystal structure as a function of simulation time are compared at the temperature 310 K (A) and 400 K (B). (TIF) [file pone.0064886.s005.tif]
